# Possible origins and implications of atypical morphologies and domestication-like traits in wild golden jackals (*Canis aureus*)

**DOI:** 10.1038/s41598-023-34533-w

**Published:** 2023-05-06

**Authors:** Ayelet Barash, Shlomo Preiss-Bloom, Yossy Machluf, Elena Fabbri, Dan Malkinson, Edoardo Velli, Nadia Mucci, Alon Barash, Romolo Caniglia, Tamar Dayan, Yaron Dekel

**Affiliations:** 1 School of Zoology and The Steinhardt Museum of Natural History, Tel Aviv University, Tel Aviv, Israel; 2 Unit of Agrigenomics, Shamir Research Institute, University of Haifa, 1290000, Kazerin, Israel; 3 The Cheryl Spencer Department of Nursing and The Cheryl Spencer Institute of Nursing Research, University of Haifa, 3498838 Haifa, Israel; 4 Unit for Conservation Genetics (BIO‑CGE), Italian Institute for Environmental Protection and Research (ISPRA), Via Cà Fornacetta 9, Ozzano dell’Emilia, 40064 Bologna, Italy; 5 Department of Geography and Environmental Studies, University of Haifa, 3498838 Haifa, Israel; 6 The Azrieli Faculty of Medicine, Bar Ilan University, 8 Henrietta Szold St, Safed, Israel

**Keywords:** Evolutionary biology, Genetic markers, Evolutionary ecology

## Abstract

Deciphering the origins of phenotypic variations in natural animal populations is a challenging topic for evolutionary and conservation biologists. Atypical morphologies in mammals are usually attributed to interspecific hybridisation or *de-novo* mutations. Here we report the case of four golden jackals (*Canis aureus*), that were observed during a camera-trapping wildlife survey in Northern Israel, displaying anomalous morphological traits, such as white patches, an upturned tail, and long thick fur which resemble features of domesticated mammals. Another individual was culled under permit and was genetically and morphologically examined. Paternal and nuclear genetic profiles, as well as geometric morphometric data, identified this individual as a golden jackal rather than a recent dog/wolf-jackal hybrid. Its maternal haplotype suggested past introgression of African wolf (*Canis lupaster*) mitochondrial DNA, as previously documented in other jackals from Israel. When viewed in the context of the jackal as an overabundant species in Israel, the rural nature of the surveyed area, the abundance of anthropogenic waste, and molecular and morphological findings, the possibility of an individual presenting incipient stages of domestication should also be considered.

## Introduction

The golden jackal is a widespread medium-sized canid prospering in various habitats from semi-deserts and forests to agricultural and urban areas. Fossil records indicate that the species has existed in the Mediterranean region since the Holocene and that local populations have mixed with jackals coming from India^1^. Golden jackals are endemic not only in the Middle East but also in southern Asia and southern Europe, and to the best of our knowledge, have been so for millennia. In the mid-nineteenth century, the jackal population started expanding to south-eastern Europe, and since the mid-twentieth century, it has expanded further northward and westward. Therefore, golden jackal numbers have increased steadily in recent years^2–6^.

From the genetic point of view, the current golden jackal population in Israel is considered of Eurasian origin (*Canis aureus*)^7^. However, some studies have also documented a subset of Israeli jackals presenting a nuclear and mitochondrial DNA haplotype shared with the recently recognized African wolf (*Canis lupaster*, previously considered a possible subspecies of golden jackal)^7,8^, a new monophyletic taxon, more closely related to the grey wolf than to the golden jackal clade. Israeli jackal populations carrying this different mitochondrial DNA (mtDNA) haplotype display a similar morphology, at least to the naked eye, and no ecological niche differentiation is evident. Of note, not only genetic data^7,9–13^ but also recent skull morphology evidence^12,14,15^ supports evolutionary differentiation between Eurasian golden jackals and African wolves, which represent distinct lineages^8^. Since the distribution range of the African wolf is limited to the African continent^7–9,16^, these findings may be interpreted as the legacy of ancient gene introgressions that occurred during the Neolithic expansion of such species from Africa to the Arabian Peninsula, likely following human migrations^17^, highlighting the importance of historical admixture and post-speciation interspecific gene flow, including potential hybridization (also with domestic dogs), in shaping the evolutionary histories of canids^7–9,16^. Therefore, from here on, for the sake of simplicity, the Israeli golden jackal population will be considered to comprise both mtDNA haplotypes (African wolf and golden jackal), unless specified differently.

In Israel, golden jackals were nearly extirpated in the 1960s as part of an eradication program to control rabies^18^, just as they were overhunted and eradicated in Europe 40 years ago^19^. Since then, they have repopulated Israel with numbers rapidly increasing in many regions across the country, particularly in the Golan Heights.

The Golan Heights is a sparsely human-populated rural area of ca. 1200 km^2^, comprising pasturelands and intensive agriculture zones, representing a Mediterranean biodiversity hotspot, that hosts endangered plant communities, migratory birds and mammals^20^. Among the mammals, the jackal population in the Golan Heights attracts particular management interest. Indeed, from the 1970s the jackal population density increased to 2.5 (end of 1980s) and to 12–24 (2010s) individuals per km^2^^18,20,21^, greatly outnumbering other wild carnivores such as wolves, by a ratio of up to 100:1; thus, the golden jackal is now considered an overabundant species^22^. This rapid and widespread ongoing population explosion, attributable to the opportunistic nature of the species, must be carefully monitored and actively managed since it causes extensive damage in agriculture, to both animal and crop production^18,20^. Additionally, the proximity of jackals to human surroundings can also favour interactions with guard and herding dogs, potentially increasing the chance for anthropogenic hybridisation events, as there are no reproductive barriers between them.

Several studies on interspecific hybridisation in mammals have shown that the effects of admixture and subsequent back-crossings with one of the parental ancestors can be unpredictable, sometimes leading to the appearance of unique atypical morphologies^23,24^. For example, in Croatia, anomalous phenotypes recently documented in some golden jackals—displaying partial dog-like morphology—have been genetically confirmed to be a consequence of anthropogenic hybridisation with dogs^25^. Another case of a jackal showing a peculiar coat colour pattern was recently reported in India^23^, yet its origin remains unknown since it was not possible to ascertain whether the coat colour pattern could be attributable to hybridisation or *de-novo* mutations.

There is a large body of work regarding the body shape of mammalian hybrids^26–28^, and its implications for speciation processes and species–conservation importance. While some studies have suggested that hybrids display a morphological resemblance to one of their paternal species^29^, most studies have revealed that hybrid specimens will either display some form of intermediate morphology or a different, unique morphology, even if one paternal ancestor contributes less than 25%^30^. It is also important to note that in many cases hybrid species display some degree of pathological condition^31–33^.

However, although most studies conducted on natural mammal populations usually link morphological variations to interspecific hybridisation or *de-novo* causal adaptive mutations, the domestication syndrome can sometimes lead to similar aesthetic modifications, including coat colour changes, drooping ears, loss of horns, leg shortening, loss of periodic hair shedding, overall smaller size (when directional selection for larger size is absent)^34–36^ and, in dogs, the upturned tail, which clearly differentiates them from wolves, following Linnaeus in his *Systema Naturae*^37^. Another domestication feature is white patches on the canid brown/grey chest, which was termed the "star gene", and was documented in the farm fox domestication experiment carried out in Novosibirsk over 60 years ago^38^.

Fifteen genes known to cause specific canine coat colour patterns regulate the pigment synthesis pathway within the hair follicle. Among them, three major genes, melanocortin 1 receptor (*MC1R*), agouti signalling protein (*ASIP*) and canine β-defensin 103 (*CBD103*), play a key role in this pathway. Products of these genes modulate the relative synthesis of eumelanin and pheomelanin, interact with each other and with the melanocyte inducing transcription factor (*MITF*), which is characterised by particular mutations associated with leucism and other multiple pathologies^39–41^. However, it is still unclear whether coat colour variation is the result of an initial human selection for tameness, or if it is a result of a causal genetic mutation that became fixed in a wild population generating an appearance less feared by humans^23,24^. Whatever the reason for these variations in coat colour, it is still widely assumed that this is the first trait that appeared in domesticated mammals^42^.

The appearance of atypical morphologies or phenotypic variations among natural animal populations is a challenging research topic. Determining the relative importance of genetic and environmental factors that modify or lead to certain traits may reveal the evolutionary dynamics that shape populations as well as ascertain whether human-driven selection is involved^43^. Phenotypic variation among canids has been mainly attributed to inter-taxon hybridisation or de-novo mutations, yet interactions with human surroundings that may lead to a domestication-like phenotype cannot be excluded. These alternative hypotheses, partially supported by recent evidence among golden jackals, may explain certain atypical morphologies among wild jackal individuals.

Therefore, the aims of this study were to monitor and portray anomalous morphology among golden jackals in order to better understand the origin of such atypical morphology, namely, to examine whether anthropogenic hybridisation, de-novo mutations or the domestication process are involved. The Golan Heights provides a unique opportunity to address these research questions since it allows us to carefully follow the dynamics of the abundant population of golden jackals^22^, detecting diverse and rare morphological variations among individuals. It also allowed us to monitor the potential effects of the close proximity of wild animals to the human population, focusing on possible hybridisation with dogs as well as on likely domestication events, whether passive (self) or active (driven partially or fully by humans)^44,45^. Thus, a camera-trapping ecological survey was established, jackals displaying atypical morphology were observed, and samples of one such individual were genetically and morphologically analysed to determine its parental lineage and population structure, which may shed light on the origin of this phenomenon.

## Results

### Observing atypical morphology in jackals

**Figure 1:**
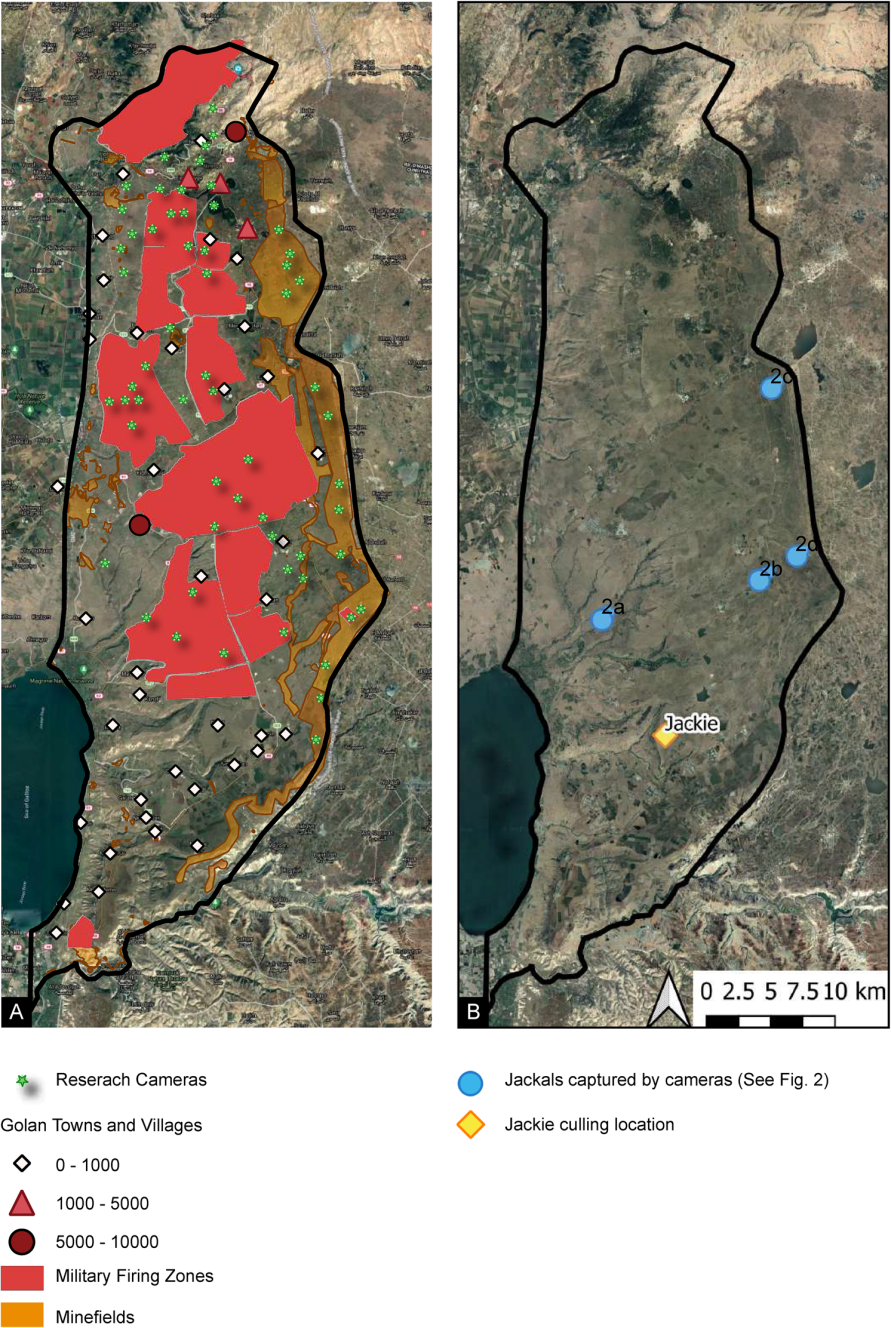
Golan Heights maps with: (**A**) land use details and camera locations; (**B**) locations of jackals displaying domestication features shown in Fig. 2. Blue circles: camera trap locations. '2a' refers to the subject presented in Fig. 2A, captured by Camera 7, Yehudiya Forest Nature Reserve: 32.9275,35.6938; '2b' refers to the subject presented in Fig. 2B, captured by Camera 3, Keshet area: 32.9545,35.8246; '2c' refers to the subject presented in Fig. 2C, captured by Camera 25, Minefield Ein Zivan area: 33.0891,35.8357; '2d' refers to the subject presented in Fig. 2D, captured by Camera 21, Minefield Keshet area: 32.9712,35.8561. The yellow diamond indicates the culling location of Jackie presented in Fig. 2E and Fig. 2E', Natur village limits: 32.846225,35.745761. The maps were created using QGIS (version 3.10, https://download.qgis.org). Satellite imagery was provided by Google Satellite (https://qms.nextgis.com/geoservices/678/) and incorporated into QGIS using the QuickMapServices plugin (https://plugins.qgis.org/plugins/quick_map_services/).

**Figure 2:**
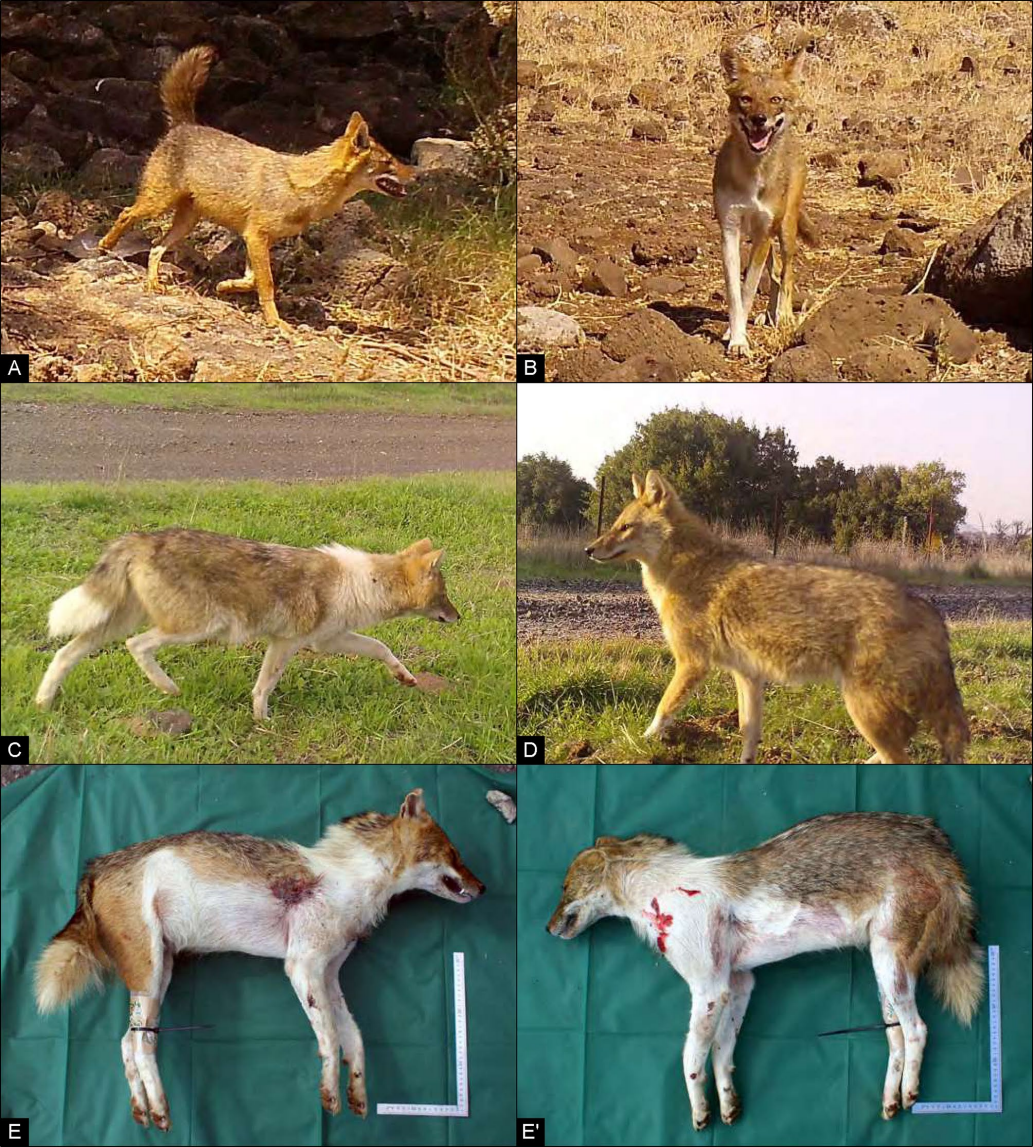
Golden jackals with domestication features. (**A**) upturned tail, (**B**) white colouration on chest and right leg, (**C**) long fur and broad white colouration, (**D**) long fur. (**E**) Right side of culled specimen carcass (Jackie), blade of steel square ruler is 30 cm, tongue of ruler is 15 cm; (**E**′) left side of specimen.

A camera-trapping wildlife survey was conducted in the Golan Heights to monitor the golden jackal population. We evaluated surveillance photographs from 60 trail cameras, dispersed across most of the study area at 12 sites of ~ 30 km^2^ each (Fig. 1A, see Supplementary Table 1 for GPS geographic coordinates of cameras and environmental characteristics). More than one million images were taken, and golden jackals—based solely on morphological evidence at this stage—were identified in 64,947 images captured in 11,959 independent observations. Among the collected images, four golden jackals observed in the central-southern sector of the study area (Fig. 1B) presented atypical morphological characteristics, such as white patches, an upturned tail and long thick fur (Fig. 2, panels A–D).

Another male jackal with anomalous features including white patches on abdomen, legs, neck and tail, in an asymmetrical pattern, was shot by the Israel Nature and Parks Authority (INPA) as part of a culling program carried out in domestic herd zones (Fig. 2, panels E–E′). This individual, hereafter designated ‘Jackie’, was morphologically examined, and a muscular tissue sample and the entire skull were collected for molecular analyses and skull geometric morphometric analyses, respectively.

### Jackie's maternal and paternal lineages

To establish Jackie’s maternal and paternal origins, the mitochondrial DNA control region (mtDNA CR, a 490-bp fragment) and a zinc-finger protein gene on the Y chromosome (ZFY-intron, where the 30-bp deletion in *Canis aureus*, compared to *Canis lupus familiaris*, the domestic dog, is located in the middle of the 455-bp fragment) were sequenced, respectively, to provide direct genetic evidence on the presence of traces of anthropogenic hybridisation, and if detected, an indication as to the possible directionality of the phenomenon.

**Figure 3:**
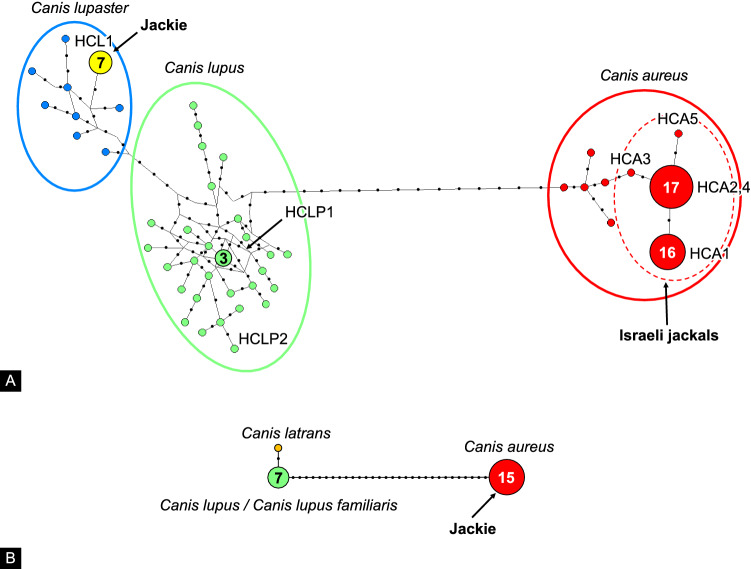
Median-joining (MJ) networks showing (**A**) mtDNA CR and (**B**) ZFY haplotype evolutionary relationships. Haplotype circle sizes are proportional to the number of individuals represented by each haplotype utilized in this analyses and colour coded according to the different *Canis* taxa analysed. The mtDNA CR haplotype codes detected in this study are shown as indicated in Supplementary Table 2. Small black dots on internodes indicate the number of nucleotide substitutions between haplotypes. (**A**) The mtDNA CR MJ network was built using haplotypes detected in this study and, for comparative purposes, 36 European *Canis lupus lupus* (light green circle), nine Eurasian *Canis aureus* (red circle) and eight African *Canis lupaster* (light blue circle) homologous sequences, retrieved from GENBANK, corresponding to a total of 47 unique *Canis* mtDNA CR haplotypes. (**B**) The ZFY MJ network was built using the unique jackal (*Canis aureus;* red circle) haplotype found in 15 samples (including Jackie), analyzed in this study. For comparative purposes, the analysis also included a coyote (*Canis latrans;* orange circle), five worldwide gray wolf (*Canis lupus;* light green circle) and two dog (*Canis lupus familiaris;* light green circle) ZFY homologous sequences, retrieved from GENBANK, corresponding to a total of three unique *Canis* ZFY haplotypes.

To establish maternal origin, 42 golden jackal samples (30 males, 12 females, see Supplementary Table 2 for specimen details), were sequenced at the mtDNA CR region (GenBank accession number NC_002008.4)^25^. Thirty-five subjects shared five mtDNA haplotypes typically found in Eurasian golden jackal (*Canis aureus*) populations (GenBank accession number NC_067757.1), which were named in this study HCA1 to HCA5, and seven subjects, including Jackie, carried the African wolf (*Canis anthus/lupaster*) mtDNA haplotype (GenBank accession number KT378607.1), named in this study HCL1 (see Fig. 3A and Supplementary Table 2).

The mtDNA CR median-joining (MJ) network (Fig. 3A) topology unambiguously separates *Canis aureus* (red circle), *Canis lupus* (light green circle) and *Canis lupaster* (light blue circle) clades, confirming that the African wolves are more closely related to the wolf than to the jackal lineage. Haplotype HCL1, which was found in seven samples including Jackie (yellow circle), clearly clustered with the *Canis lupaster* clade, whereas the five haplotypes (HCA1-5) detected in the other 35 jackal samples of this study (included in the dashed red circle) clearly clustered within other Eurasian *Canis aureus* haplotypes. The two haplotypes detected in the four samples belonging to *Canis lupus pallipes*, HCLP1-2, grouped, as expected, within the wolf clade.

To determine their paternal origin, DNA samples from 15 male golden jackals (see Fig. 3B and Supplementary Table 2), including Jackie, were sequenced at the diagnostic segment of the ZFY-intron, which distinguishes jackals from dogs. All samples matched the typical Eurasian *Canis aureus* Y-chromosome sequence (accession number KF021269.1)^46^. Two dogs were sequenced as positive controls and matched the dog Y-chromosome sequence (accession number KP081776.1).

The ZFY MJ network (Fig. 3B) topology clearly separates *Canis aureus* (red circle), *Canis lupus/Canis lupus familiaris* (light green circle) and *Canis latrans* (orange circle) clades. All male jackals from the study area, including Jackie, showed the deletion of 30-bp, typical of this taxon, on the on the Y chromosome.

Hence, the paternal nuclear genetic results provide evidence of Jackie’s golden jackal lineage, whereas maternal genetic results suggest historical African wolf introgression, in agreement with previous studies, attesting to the presence of historical African wolf mtDNA introgression in the study area^7^. Nevertheless, the possibility that Jackie is a hybrid between the Eurasian golden jackal and the African wolf cannot be ruled out. Either way, these findings cannot directly explain the atypical morphological characteristics of Jackie, whose appearance partially resembles that of the domestic dog.

### Population structure and admixture detection analyses

**Figure 4:**
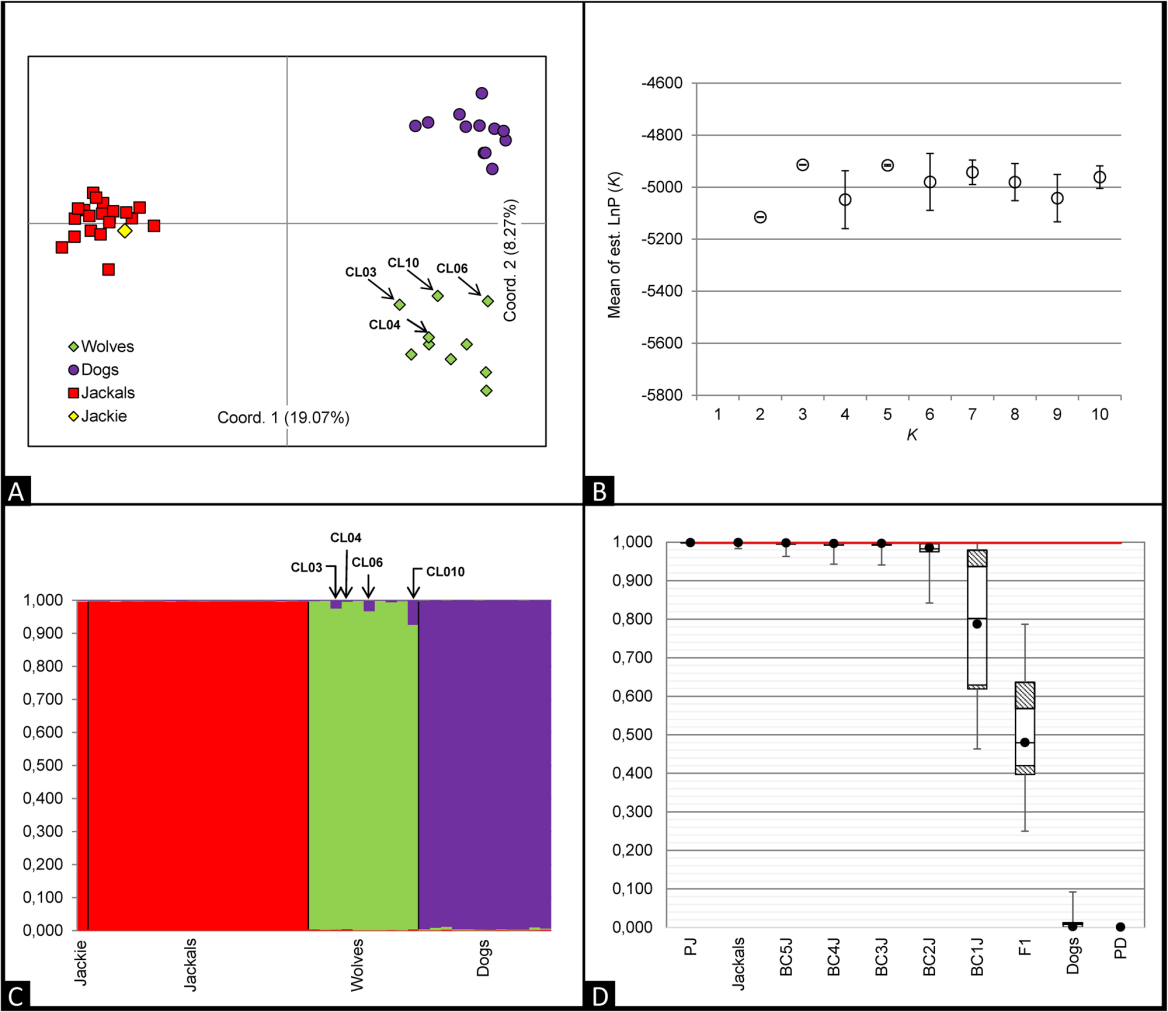
(**A**) Explorative Principal Coordinates Analysis (PCoA) performed using the 38-STR genotypes of 10 wolves (light green diamonds), 12 dogs (violet dots), 20 jackals (red squares), and Jackie (yellow diamond). Coordinate I (explaining 19% of the total genetic variability) clearly separates jackals (left side) from wolves and dogs (right side); while Coordinate II (explaining 8% of the total genetic variability) clearly separates wolves (top right) from dogs (bottom right). (**B**) Estimated posterior probability LnP(*K*) of the *K* genetic clusters from 1 to 10. (**C**) Bar plot of individual *q*i-values obtained through Bayesian model-based clustering procedures performed using the 38-STR genotypes of 10 wolves, 12 dogs, 20 jackals, and Jackie. Each individual is represented by a vertical line partitioned into coloured segments, whose length is proportional to the individual coefficients of membership (*q*i) in the jackal, dog and wolf clusters. (**D**) Box plots of individual *q*i-values (on the y-axis) observed in parental and simulated 38-STR genotypes (on the x-axis) estimated from the Bayesian model-based clustering analyses performed in Structure, assuming *K* = 2 clusters and using the “Admixture” and “Independent allele frequencies” models. Solid white boxes include 90% of the observed data values. Dashed grey boxes contain the 5th percentile and the 95th percentile of the observed data values. Black dots indicate mean data values. Middle transversal lines inside boxes show median data values (the 50th percentile). Box plot whiskers include the ranges of the confidence intervals. The red line indicates the *qi*-value (*q*i = 0.999 CI = 0.995–1.000) of the Jackal cluster observed in Jackie. PJ, simulated parental jackal genotypes; PD, simulated parental dog genotypes; BCJ, simulated backcrosses between F1 and parental jackal genotypes.

The genetic population structure and potential signs of inter-taxon admixture in the Israeli canids were assessed by genotyping the 43 collected samples (10 Israeli wolves, *Canis lupus pallipes*, CL; 12 domestic dogs, *Canis lupus familiaris*, CF from breeds commonly used in the Golan Heights by ranch owners; 20 Israeli golden jackals, *Canis aureus*, CA; and Jackie) at 38 autosomal microsatellite markers (STRs). The three canid groups were well-separated in the exploratory principal coordinate analysis (PCoA), with three wolves (CL03, CL06 and CL10) clearly clustering near the dogs and Jackie fully overlapping with the jackals (Fig. 4A). Multivariate analyses were strongly supported by the Bayesian model-based clustering procedures implemented in the software STRUCTURE which showed increasing rates in the estimated posterior probability LnP(*K*) of the clusters up to *K* = 3 (Fig. 4B). At *K* = 3, Israeli dogs (*Q*1 = 0.994, CI = 0.964–1.000), Israeli wolves (*Q*2 = 0.981, CI = 0.900–1.000) and Israeli jackals (*Q*3 = 0.997, CI = 0.984–1.000) clustered separately, with no sign of dog or wolf admixture in the jackal samples (Fig. 4C). Consistent with the PCoA results, three wolves (CL03, CL06 and CL10) showed significant signs of dog ancestry, ranging from 4 to 7%, at the STRs. Jackie was unambiguously assigned to the jackal cluster with a proportion of posterior probability *q*j = 0.996 (CI = 0.975–1.000). Sample gender was molecularly assessed and always consistent with individual morphological descriptions. One wolf sample (CL04) showed a Y-haplotype shared with three of the analysed Israeli dogs, although this subject was entirely assigned to the wolf cluster showing a *q*w = 0.993 (CI = 0.956–1.000) at the STRs. The four Y-chromosome STRs were confirmed to not be amplifiable in jackals^47^. Furthermore, none of the 43 analysed canid DNA samples, including the one belonging to Jackie, revealed the presence of the deletion of 3 base pairs in the *CBD103* gene which usually determines a melanistic coat colour in canids^48^.

Neither dog nor wolf private alleles were detected in Jackie’s genotype. Its genetic profile showed 13 private jackal alleles (distributed in ten loci), 12 alleles (in 12 loci) shared by jackals and wolves and two alleles (in two distinct loci) not detected in any other analysed sample.

Additional admixture analyses performed on empirical and simulated genotypes (ten first generation hybrids and 100 individuals belonging to the first ten generations of backcrossing with jackal ancestors) confirmed Jackie to be a jackal, excluding any presence of domestic dog ancestry at the analysed autosomal loci more recent than the fifth generation of backcrossing (Fig. 4D). Therefore, though our reference populations did not include a subset of African wolf genotypes, both empirical and simulated results from the autosomal STR Bayesian model-based clustering procedures led us to rule out Jackie as an admixed individual between the Eurasian golden jackal and the African wolf since we observed a full assignment of Jackie’s multilocus profile to the golden jackal genetic cluster, with no other significant genetic canid components which we eventually would expect due to the closer evolutionary relationships between the African wolf and the grey wolf lineages.

### Skull morphology

**Figure 5:**
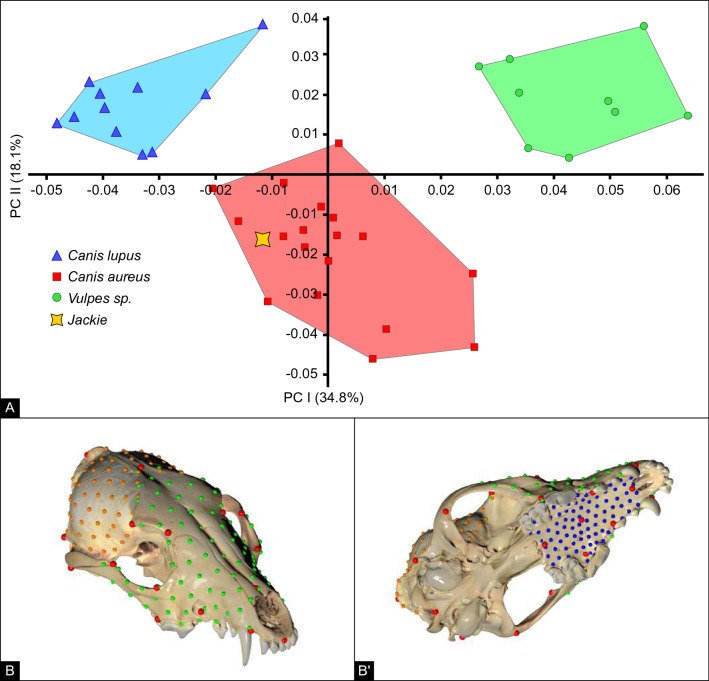
(**A**) Principal component analysis results for GM shape analysis. Jackie (Yellow star) falls well within the *Canis aureus/anthus* clade. Green circles: foxes (*Vulpes vulpes*); red squares: golden jackals (*Canis aureus/anthus*); Blue triangles: wolves (*Canis lupus pallipes*). (**B**) Superior view of landmarks used in this morphometric analysis placed on Jackie's skull. (**B** and **B**′) Inferior view. Red dots indicate osteometric landmarks. Green: facial semi landmarks; orange: neurocranium; blue: hard palate. Anatomic landmarks are provided in Supplementary Table 3.

To examine Jackie’s skull morphology and establish whether it displays any signs of hybridisation or developmental pathologies, we performed a geometric morphometrics (GM) shape analysis. Principal component analysis (PCA), based on this GM shape analysis, including data from 11 Israeli wolves, *Canis lupus pallipes*, CL; 18 Israeli golden jackals, *Canis aureus*, CA; 9 Israeli foxes, *Vulpes vulpes* and Jackie, revealed a clear distinction between the sampled canid skulls. The first (34.5%) and second (18.1%) components clearly separated foxes, grey wolves, and golden jackals. No clear differentiation was detected between Eurasian jackals and African jackals in skull size, shape, or proportion. In the GM analyses Jackie clearly clustered among the jackals, being indistinguishable from the other jackals (Fig. 5A).

Additionally, osteometric landmarks and semi-landmarks on Jackie’s skull did not display any degree of a pathological condition (Fig. 5B, B′ and Supplementary Table 3). Domestic dog skulls were not included in these morphometric analyses due to the large variation among breeds that would skew the results and probably lump all wild canid groups together.

## Discussion

Based on a widespread ecological camera-trapping study carried out in the Golan Heights, a biodiversity hotspot in the Mediterranean bio-geographic region, with assistance from an artificial image screening approach, we obtained several reliable field observations that allowed us to identify five wild golden jackals (*Canis aureus*) in the study area displaying atypical morphological features, some of which are compatible with the domestication syndrome. One of these individuals (named Jackie) was legally culled under a permit released according to Israeli wildlife control laws, allowing us to examine it in detail and try to understand the possible origins of its uniqueness through a multidisciplinary approach based on genetic and geometric morphometric analyses. Both uniparental (mtDNA control region and a ZFY fragment) and bi-parental (38 autosomal STRs) molecular markers, as well as skull morphometric analyses, clearly identified Jackie as a golden jackal showing traces of the historical mtDNA introgression from the African wolf (*Canis lupaster*) rather than a recent dog/wolf-jackal hybrid. Importantly, Jackie's mitochondrial-nuclear discordance should not contribute to its anomalous phenotypic features since: (1) both wild golden jackals and African wolves share very similar morphologies but never including the unique features observed in Jackie; and (2) other individuals showing the same genetic discordance were found to be morphologically indistinguishable from the wild golden jackal phenotype^7^.Therefore, in light of the obtained results, three possible alternative scenarios may be proposed to interpret Jackie's atypical morphology.

According to the first hypothetical scenario, the morphological anomalies observed in Jackie, and in the other recorded morphologically atypical jackals, might be the consequence of anthropogenic hybridisation, as previously described for other jackal-dog admixture cases showing unusual phenotypic traits in Croatia^25^. Indeed, a high number of wild jackals and the widespread presence of herding dogs moving freely within jackal territories have been repeatedly documented in the Golan Heights, thus potentially increasing the probability of interspecific admixture. However, our preliminary genetic and morphometric analyses, carried out on Jackie’s DNA and skull, showed no apparent evidence for such a hybridisation event for at least the last five generations. These findings suggest that the particular morphology of Jackie might be due to ancient introgressions of domestic dog alleles, that were almost completely eroded after a few backcrossing generations, leaving traces of dog ancestry only at particular genomic regions or in linkage with multiple or particular coat colour and other morphologically associated genes, and thus not detectable at the few uniparental and autosomal loci we analysed. Future genome-wide studies, based on ancestral chromosomal block reconstruction methods^49,50^ could help identify possible dog-derived regions along Jackie's genome, potentially the reason for the observed morphological anomalies, as previously conducted in North American grey wolves to detect a functional 3-bp melanistic deletion along chromosome 16, presumably introgressed via historical admixture with dogs^51,52^, or in North American bison (*Bison bison*) populations to identify cattle haplotypes introgressed in their genomes over the last 200 years^53^.

According to the second hypothetical scenario, the uncommon features observed in the Golden Heights jackals might be the expression of natural variation produced by *de-novo* causal mutations at genes related to coat colour determination and other morphological traits, i.e., not due to past admixture with dogs. Of note, Shameer and colleagues suggested that "the coat colour variation [either due to cross-breeding with dogs or acquired mutations] helps camouflage the jackals as free-ranging dogs, thus possibly helping them escape from anthropogenic threats" implying that this is an adaptive trait. Indeed, none of the analysed samples showed traces of dog ancestry at the only morphology-linked gene we investigated, *CBD103*, which was tested as part of the STR panel applied for the individual genotyping. However, it is currently known that multiple genes are simultaneously involved in canine morphology and coat colour patterns^39–41^ and their combined effects on the expressed phenotype and colouration can also determine direct or indirect adaptive advantages in different ecological contexts. In fact, a number of selection studies have highlighted latitudinal camouflage advantages in densely forested areas or a functional resistance to pathogen infections in North American and European black canids carrying the 3-bp deletion at the β-Defensin gene^52,54–56^, as well as positive selection and increased fitness rates in arctic habitats for lighter coloured Greenland and Tibetan wolves^39^. The de-novo mutations potentially responsible for the lighter and white spotted coat colours observed in Jackie and in the other four Golan Heights jackals might have randomly originated in a single individual and then been transmitted to the subsequent generations by circulating among a few related animals, potentially conferring onto them an adaptive advantage in a Mediterranean semi-desert with sparse arboreal vegetation. All these findings might support this second scenario of multiple *de-novo* mutations, which after their occurrence and their random independent inheritance in the course of mating between carrier individuals would not confer a single anomalous phenotypic trait, but rather, through pleiotropic effects, multiple features, such as those observed in the atypical animals of the study area. However, the preliminary molecular analyses conducted in this study do not allow us to confirm or reject such a hypothesis since, despite the 3-bp melanistic deletion at the *CBD103* gene, no other genes likely related to coat colouration and morphology have been exhaustively typed^52^. Therefore, future genome-wide studies could be conducted to analyse the Golan Heights canids using specific targeted DNA capture arrays^57^ ad hoc designed for candidate morphology and coat colour genes potentially under positive selection, thus identifying possible islands of adaptive variation and environmentally driven functional variants related to local adaptation. Moreover, other future research projects planned to monitor the dynamics of Golan Heights canid population, based on the genotyping of other atypical wild individuals legally culled or found dead in the study area, could surely contribute to clarifying the existing kinship relationships among them and, therefore, shed light on the spatio-temporal origin and spread of such anomalies.

However, the morphological anomalies observed in the study area i.e., white patches, thick long fur and an upturned tail (Fig. 2), together with the rarity of the recorded events—only five wild individuals displaying different uncommon features over approximately 12,000 independent sightings—suggest a third hypothesis, which does not contradict the previous scenario. According to this hypothesis, wild jackals may undergo an incipient partial self-domestication process, since the observed uncommon features seem to be coherent with the characteristic phenotypic changes, including coat colour variation, emerging during the preliminary stages of domestication under directional selection^24^. The demographic explosion of the jackal population in Israel, which is also favouring its spread into anthropic settlements, as well as the opportunistic nature of the species that drives it to exploit human wastes, are conditions that could facilitate not only anthropogenic hybridisation but also initiate a possible process of self-domestication in the Golan Heights, similar to what has been hypothesized for the African wolf during the Neolithic agricultural revolution in North Africa^17^. The latter scenario constitutes a unique evolutionary and cultural phenomenon in which wild animals can be modelled into their domesticated forms, for the mutual benefit of both animals and humans^58–61^.

Several theories and schemes have been proposed to describe the intriguing process of domestication, although it could be much more complex than hitherto hypothesised^60,62^, with multiple independent events involving different populations of the same taxon under different ecological conditions^63,64^. Indeed, the jury is still out on whether wild mammals slowly transformed into their domesticated form around the developing human surroundings or whether our ancestors actively captured and tamed the first individuals who later developed into domesticated animals^65,66^. In the case of golden jackals, our results suggest the former, as the anthropogenic niche itself seems to be the driving force, whether coat colour variation is the result of a genetic mutation favoured by humans, or of an initial selection for tameness. Whether these are voluntary or involuntary selections on specific behavioural genes, that could mediate the loss of fear towards humans, is still hotly debated. The anomalous morphological features, convergent with domestic dog phenotypes, observed in the Golan Heights golden jackals might confer onto them bolder behaviour and, thus, better access to human food sources, and might provide them indirect advantages in semi-anthropogenic landscapes, making such suspected proto-domesticated animals more accepted and less feared by humans^24,65,67^.

The scenarios proposed for interpreting the observed morphological anomalies in Jackie and in the other four jackals of the Golan Heights appear to be equally possible since the preliminary results we obtained do not allow us to draw definitive conclusions. Furthermore, a series of limitations associated with the present study must be taken into consideration: (1) only one of the observed individuals showing the uncommon features was genetically and morphologically analysed. Since ecological surveys in the study area are usually based on photo-trapping techniques, genetic analyses on non-invasively collected samples can rarely be directly associated with the images captured; (2) morphometric analyses could not include any dogs due to the vast variability among breeds; (3) in the absence of reliable molecular markers directly associated with domestication^68^, genetic analyses were performed using a few markers, which, although highly differentiating between dogs, wolves and jackals, represent only a moderately resolved snapshot of the non-coding variability observable within the whole canine genome.

Future whole-genome, transcriptome, genome-wide marker and gene ontology comparative analyses conducted on wild and domestic Israeli canids, including those with suspected dog-derived or domestication features, could help to explain the unique morphology of Jackie, and definitively clarify whether such a fascinating animal is the result of an ancient jackal-dog anthropogenic admixture, the manifestation of phenotypically expressed *de-novo* causal mutations linked to local adaptive variation, or the possible product of an ongoing partial self-domestication process. Will the discovery of ‘Jackie’ be another milestone in deciphering this fascinating process of animal and plant domestication? Further exploration in the wild, in Israel and other locations with similar ecological contexts, might reveal if this is indeed the case.

## Methods

### Camera surveillance and spatial analyses

Camera surveillance took place between August and December 2020, at twelve ~ 30 km^2^ sites located in the central and northern Golan Heights (Supplementary Table 1). The sampling sites designated for this study belonged to four different land-use categories: “high culling risk”, “low culling risk”, nature reserves and minefields. Within each site, five Browning DARK OPS HD PRO X trail cameras were randomly installed along paths walked by animals, with a minimum of 1 km between each two cameras. Cameras were installed at a height of 0.5 m off the ground and a 45° angle to the path. At each installation the following data were collected using structured questionnaires in the Epicollect5 mobile application: coordinates, date and time, installer/s, site name, camera ID, memory card ID, camera sensitivity settings, vegetation description, type and width of path, fencing, livestock presence, agriculture intensity, passability behind camera, and picture. Memory cards were collected and replaced at two intervals over the course of this study, during which batteries were replaced as needed and the following data were recorded: camera condition (in place/askew/on ground/missing), card fullness, and camera sensitivity settings.

Approximately 1 million images were gathered in total. Images collected by false triggers caused by wind and moving vegetation were filtered out using Microsoft’s Artificial Intelligence MegaDetector model with a threshold of 0.8. After filtering the false triggered images, the remaining 351,804 images of animals, humans and vehicles were reviewed manually in Camelot camera-trap data management software^69^. In each of these images, we identified the species, number of individuals and developmental stages. The above data in Camelot were used to produce two occupancy matrix reports—one showing the number of independent sightings of a species per camera trap, and the other showing whether a species was present at a camera trap on a certain day.

Spatial analysis was performed in QGIS 3. The following land use layers were collected and fitted within a 10 km buffer zone around the Golan Heights to be used as spatial covariates in this study: minefields, nature reserves, military firing zones, built-up areas, orchards, agricultural fields, open spaces, perennial water sources, and cattle structures. Pixel size was designated as 25 m^2^.

### Biological sample collection and DNA extraction

Tissue samples were obtained from 52 golden jackals from different regions in Israel (33 males, 19 females, including Jackie (Supplementary Table 2)). The DNA samples from the golden jackals were extracted as follows: DNA from muscular tissues (culled wild specimens) was extracted using the Qiagen DNeasy Blood and Tissue Kit protocol and was further assessed for quality (260/280 nm) and quantity (> 30 ng) with the BioTek, Synergy H1 (Bad Friedrichshall, Germany). The biological samples used in this research were collected, packed, stored and treated following the required standards and with appropriate permissions from all relevant parties. Some samples were taken from the Steinhardt Museum of Natural History (SMNH) collection, others were collected by INPA rangers from roadkill and culling. Following Israeli law, the samples collected from animals complied with institutional and national guidelines. The study protocol of obtaining samples and analysing the findings of the genetic tests for research purposes was approved by the INPA (Permit Number: 42429).

### Jackie sampling

The jackal with atypical morphological features (Jackie) was shot on June 21st, 2021. Three different muscular tissue samples were collected into three different Eppendorf tubes. The DNA was extracted from each sample separately, and Polymerase Chain Reaction (PCR) amplification was independently performed on each sample for the ZFY-intron and mitochondrial CR markers (see below). All six PCR products were sequenced at Macrogen Europe, Amsterdam, Netherlands. The carcass was later sent to the SMNH (assigned number 17268) at Tel- Aviv University for storage of tissue samples and the skull.

### DNA amplification and sequencing

Primers for the ZFY-intron region^46^ and mitochondrial CR^25^ were designed with the Primer3plus tool (https://www.bioinformatics.nl/cgi-bin/primer3plus/primer3plus.cgi) and synthesised by Hylabs, Rehovot, Israel. The PCR reactions were performed on each marker with its designed primers using the HotStart HiFidelity Polymerase Kit protocol^70^ or the Qiagen LongRange PCR Kit^71^. Results were analysed with the QIAxcel Advanced System (Qiagen Inc., Hilden, Germany). The PCR products were sent for purification and Sanger sequencing to Macrogen Europe, Amsterdam, Netherlands.

### Establishing parental lineage

Mitochondrial CR amplification and sequencing protocols were conducted according to Galov et al*.*^25^. Five grey wolves and two dogs were sequenced as positive controls. The resulting sequences were validated in vitro with the Sanger sequencing method and the NCBI nucleotide BLAST tool (https://blast.ncbi.nlm.nih.gov/Blast.cgi?PAGE_TYPE=BlastSearch) was used for the alignment of sequences to relevant accessions (AF098116.1 for grey wolves and KU290854.1 for dogs).

The ZFY-intron amplification and sequencing protocols were conducted according to Tsubouchi et al*.*^46^. To determine paternal origin, DNA samples of 15 male golden jackals, including Jackie, were sequenced using a Canid-specific PCR-based ZFY-intron method according to which golden jackals yield a shorter amplicon due to a 30-bp deletion in comparison to dogs. Two dogs were sequenced as positive controls and matched the dog Y-chromosome sequence (accession number KP081776.1).

Two median-joining (MJ) networks were reconstructed by the software NETWORK v4.6^72^ to investigate inter-taxon haplotype relationships at both the maternal and paternal markers.

The mtDNA CR network was built using the two wolf (*Canis lupus pallipes*), five jackal (*Canis aureus*) and one African wolf (*Canis lupaster*) mtDNA CR haplotypes detected in this study. For comparative purposes, the analysis also included 36 *Canis lupus lupus* from Europe^73,74^ (accession numbers: KY549989-KY550013), nine *Canis aureus* (seven from India^67^; one from Europe^47^; one from Turkey: accession numbers: KT343787.1, KT343788.1, KT343790.1, KT343789.1, KT343791.1, KT343794.1, KT343795.1, KF588364, NC_067757.1), eight *Canis lupaster* from Africa (accession numbers: JQ088678.1, JQ088679.1, JQ088680.1, JQ088681.1, JQ088682.1, JQ088683.1, JQ088684.1, KT378607.1) homologous sequences, retrieved from GENBANK, which were aligned, trimmed into fragments of equal length of 197 bp using the software BIOEDIT v7.1.11^75^, and collapsed using DNASP v5.10.01^76^ in a total of 47 different unique *Canis* mtDNA CR haplotypes. At 197 bp, two Israeli golden jackal haplotypes (HCA2 and HCA4) detected in this study resulted in a single equivalent haplotype.

The ZFY MJ network was built using the unique jackal (*Canis aureus*) haplotype found in 15 samples (including Jackie), analysed in this study. For comparative purposes, the analyses also included a coyote (*Canis latrans*, accession number AB622146), five worldwide wolf (*Canis lupus*, accession numbers: KT448254, KT448255, KT448256, KT448253^7^; KF021270^77^) and two dog (*Canis lupus familiaris*, accession numbers: KF021271, KP081776^77^) ZFY homologous sequences, retrieved from GENBANK, which were aligned, trimmed into fragments of equal length of 450 bp using the software BIOEDIT v7.1.11^75^, and collapsed using DNASP v5.10.01^76^ for a total of three unique *Canis* ZFY haplotypes.

### Microsatellite analyses

Microsatellite genotyping analyses were performed using an STR panel, described as follows: a total of 43 canid DNA samples, from 10 Israeli wolves (8F and 2 M), 14 Israeli dogs (7F and 7 M) and 25 Israeli golden jackals (7F and 18 M), including Jackie (CA012M), were amplified by PCR and genotyped, through a multiple-tube approach based on two independent replicated amplifications per locus per sample, at 38 unlinked autosomal canine microsatellite (STR) loci, commonly used to reconstruct individual genotypes and reliably discriminate among wolves, dogs and their first three generation hybrids through Bayesian model-based clustering procedures^73,78^. Eleven of the 38 STRs (FH2004, FH2088, FH2096, FH2137, CPH4, CPH5, CPH8, CPH9, CPH12, C20.253, C09.250) have even been applied to investigate jackal genetic structure and to detect the first evidence of jackal-dog hybridisation in central-western Europe^25,47^. Samples were also genotyped, through a multiple-tube approach, at (1) a portion of the Amelogenin marker, to molecularly determine their gender, (2) at 4 Y-chromosome STRs^79,80^ (MS34A, MS34B, MSY41A and MS41B) to determine paternal haplotypes in male individuals; and (3) at a dominant 3-bp deletion at the β-defensin *CBD103* gene (the K-*locus*) associated with black coat colour in canids^78^. All loci were amplified through nine multiplexed reactions using the QIAGEN Multiplex PCR kit (Qiagen Inc., Hilden, Germany), in a total volume of 10 µL containing: 1 µL of DNA, 5 µL of MasterMix, 1 µL of Q-solution, 0.10–0.30 µl of primers and adjusted to the final volume with RNAse-free water. Multiplexed amplifications were performed using an ABI GeneAmp©PCR System 9700, and the following thermal profile: 94 °C/15 min, 94 °C/30 s, 57 °C/90 s, 72 °C/60 s (35 cycles), followed by a final extension step of 72 °C for 10 min. The PCR products were analysed in an ABI 3130XL automated DNA sequencer and the STR allele sizes were estimated using the ABI LIZ 500 size standard and the ABI software Genemapper v.5.0. Amplification and post-amplification of muscular DNA were conducted in separate reserved rooms, adding a blank (no DNA) plus eight positive (three known wolf, two known dog and three known jackal DNA samples) controls during DNA amplifications. Consensus genotypes were assessed from the two replicates per locus performed during the multiple-tube approach, using Gimlet v.1.3.3 software^81^.

### Population structure analyses

Patterns of differentiation among groups and individuals, as well as their distribution in the genetic space, were visualised by analysing individual 38-STR genotypes through an explorative Principal Coordinate Analysis (PCoA) performed using the software GenAlEx v.6^82^. Additionally, to reconstruct the genetic structure among sampled groups and identify possible signals of admixture among them, *multilocus* individual 38-STR genotypes were assigned to their most likely taxon of origin through Bayesian model-based clustering procedures implemented in STRUCTURE v.2.3.4 software. Four independent runs were performed for *K* values ranging from 1 to 10, using 1,000,000 Markov chain Monte Carlo (MCMC) iterations, after a burn-in of 100,000 iterations, assuming no prior information (option “*usepopinfo”* not activated), and choosing the Admixture and the Independent Allele Frequency models. The highest rate of increase in the posterior probability LnP(*K*) between consecutive *K* was used to estimate the most likely number of genetic groups, *K,* at which to assess the average (*Q*i) and the individual (*q*i) proportions of membership to each different cluster. Assignment results were integrated with the information derived from uniparental (mtDNA, four Y-linked STRs) and coding (K-*locus*) markers, which were used to confirm the taxon identification or, in the case of admixed individuals, to provide the directionality of the hybridisation^78^. Based on the assignment results, the number of private alleles for each genetic cluster was computed using GenAlEx v.6 software.

The discriminating power of our marker panel to detect jackal-dog hybrids and their backcrosses in admixture analyses, and consequently its efficacy in identifying possibly old domestic dog ancestry in Jackie’s genotype, were assessed through simulations performed with HybridLab software. Reliable jackal (*q*i > 0.995) and dog (*q*i > 0.980) individual genotypes unambiguously assigned to their own cluster in assignment procedures were used to generate 10 simulated genotypes of wild (PJ) and domestic (PD) parentals, first (F1) generation hybrids, and ten backcross generations (BC1J-BC10J) with wild parentals. The simulated genotypes were then analysed in STRUCTURE software as described above, without any prior population information. The proportion of individuals correctly assigned to each class led to accurately defining the detectable level of admixture using the applied marker panel.

### Geometrics morphometrics

For geometric morphometric shape analyses, 30 key osteometric landmarks (Supplementary Table 3) were placed on each cranium (jackals n = 19; wolves n = 11; foxes n = 9), followed by 300 semi-landmarks (Fig. 5b, c) using dHal Viewbox (ver. 4.0.1.7, http://www.dhal.com/index.htm), by one of the authors (AB). Shape analyses were carried out in EVAN Toolbox (https://www.evan-society.org/): specimens were superimposed using the Procrustes method, to disregard size, and PCA was conducted, also by the EVAN Toolbox, to detect species-specificshape differences.

### Ethics statement

Performing animal experiments was approved by the institutional board (# TAU-R-1011553). This study adhered to the tenets of the permits from the Israel Nature and Parks Authority (INPA) regarding gathering of biological samples and collecting samples from road kills (INPA Permit Number: 42429), and from the Ministry of Environmental Protection regarding collection and storage of biological samples at the Steinhardt Museum of Natural History, Tel Aviv University (Permit Number H8595). We confirm that all methods were performed in accordance with the relevant national and institutional regulations, and are reported in accordance with ARRIVE guidelines for reporting animal research^83^.

## Supplementary Information

Supplementary Tables.

## Data Availability

The datasets of this study are publicly available. Genetic sequences of the mitochondrial DNA control region (mtDNA CR) and Y-chromosome segments have been deposited in the public sequence repository GenBank: accession numbers OQ189928-OQ189974 and OQ189975-OQ189989, respectively. Additionally, autosomal microsatellite marker (STRs) data has been uploaded to Dryad (see: 10.5061/dryad.pg4f4qrtx).
