# Supplementary material for: Possible origins and implications of atypical morphologies and domestication-like traits in wild golden jackals (Canis aureus)
Source: Sci Rep. 2023 May 6;13:7388. doi: 10.1038/s41598-023-34533-w (PMC10164184; doi:10.1038/s41598-023-34533-w)
Supplement: Supplementary file 1 — Supplementary Tables. [file 41598_2023_34533_MOESM1_ESM.docx]

**Supplementary Table 1:**

Location and geographic coordinates of cameras.

| Trap Station | Latitude | Longitude | Environment type | |
| --- | --- | --- | --- | --- |
|  |  |  | Primary | Secondary |
| 1 | 32.9845 | 35.7992 | Grazing Pasture |  |
| 2 | 32.9703 | 35.8227 | Grazing Pasture | Nature Reserve |
| 3 | 32.954503 | 35.824601 | Grazing Pasture |  |
| 4 | 32.9609 | 35.8119 | Grazing Pasture |  |
| 5 | 32.916702 | 35.808437 | Grazing Pasture | Military Firing Zone |
| 6 | 32.9659 | 35.6596 | Nature Reserve | Grazing Pasture |
| 7 | 32.9275 | 35.6938 | Nature Reserve | Grazing Pasture |
| 8 | 32.9139 | 35.7189 | Nature Reserve | Military Firing Zone |
| 9 | 32.9452 | 35.7324 | Nature Reserve | Military Firing Zone |
| 10 | 32.9023 | 35.7582 | Nature Reserve | Military Firing Zone |
| 11 | 32.9326 | 35.8728 | Minefield-adjacent |  |
| 12 | 32.9268 | 35.8647 | Minefield-adjacent | Military Firing Zone |
| 13 | 32.8936 | 35.8429 | Minefield-adjacent |  |
| 14 | 32.8703 | 35.8392 | Minefield-adjacent |  |
| 15 | 32.8412 | 35.8346 | Minefield-adjacent |  |
| 16 | 33.0869 | 35.7506 | Military Firing Zone | Grazing Pasture |
| 17 | 33.0977 | 35.7443 | Military Firing Zone | Grazing Pasture |
| 18 | 33.1313 | 35.7147 | Grazing Pasture | Orchard-adjacent |
| 19 | 33.1045 | 35.694 | Military Firing Zone | Grazing Pasture |
| 20 | 33.0809 | 35.7255 | Grazing Pasture |  |
| 21 | 32.9712 | 35.8561 | Minefield-adjacent |  |
| 22 | 33.002894 | 35.855016 | Minefield-adjacent |  |
| 23 | 33.020894 | 35.855401 | Minefield-adjacent |  |
| 24 | 33.068903 | 35.846359 | Minefield-adjacent | Nature Reserve |
| 25 | 33.0891 | 35.8357 | Minefield-adjacent |  |
| 26 | 33.1553 | 35.812 | Minefield-adjacent | Grazing Pasture |
| 27 | 33.175 | 35.8126 | Minefield-adjacent | Grazing Pasture |
| 28 | 33.2 | 35.8062 | Minefield-adjacent |  |
| 29 | 33.1832 | 35.8154 | Minefield-adjacent |  |
| 30 | 33.1641 | 35.8234 | Minefield-adjacent |  |
| 31 | 33.2174 | 35.7521 | Nature Reserve |  |
| 32 | 33.2124 | 35.7268 | Nature Reserve | Grazing Pasture |
| 33 | 33.1888 | 35.7303 | Nature Reserve | Grazing Pasture |
| 34 | 33.1696 | 35.7457 | Nature Reserve | Grazing Pasture, Military Firing Zone |
| 35 | 33.1857 | 35.7439 | Nature Reserve | Grazing Pasture, Military Firing Zone |
| 36 | 33.2287 | 35.7248 | Orchard-adjacent | Nature Reserve |
| 37 | 33.2293 | 35.7067 | Orchard-adjacent | Grazing Pasture |
| 38 | 33.201 | 35.7003 | Military Firing Zone | Grazing Pasture |
| 39 | 33.2315 | 35.7498 | Nature Reserve |  |
| 40 | 33.2119 | 35.716 | Military Firing Zone | Grazing Pasture |
| 41 | 33.2857 | 35.7515 | Nature Reserve | Grazing Pasture |
| 42 | 33.267 | 35.7518 | Nature Reserve | Grazing Pasture |
| 43 | 33.2593 | 35.745 | Nature Reserve | Grazing Pasture |
| 44 | 33.2489 | 35.7405 | Nature Reserve | Grazing Pasture, Orchard-adjacent |
| 45 | 33.2517 | 35.7108 | Nature Reserve | Grazing Pasture |
| 46 | 33.0902 | 35.6829 | Military Firing Zone | Grazing Pasture |
| 47 | 33.0805 | 35.6769 | Military Firing Zone | Grazing Pasture |
| 48 | 33.0798 | 35.6637 | Military Firing Zone | Grazing Pasture |
| 49 | 33.0808 | 35.6882 | Military Firing Zone | Grazing Pasture |
| 50 | 33.0629 | 35.6823 | Military Firing Zone | Grazing Pasture |
| 51 | 33.9977 | 35.7916 | Military Firing Zone | Grazing Pasture |
| 52 | 32.9914 | 35.751 | Military Firing Zone | Grazing Pasture |
| 53 | 33.0232 | 35.7526 | Military Firing Zone | Grazing Pasture |
| 54 | 33.0386 | 35.7794 | Military Firing Zone | Grazing Pasture |
| 55 | 33.0111 | 35.7705 | Military Firing Zone | Grazing Pasture |
| 56 | 33.1873 | 35.6738 | Grazing Pasture |  |
| 57 | 33.2148 | 35.675 | Grazing Pasture |  |
| 58 | 33.2312 | 35.6785 | Grazing Pasture | Orchard-adjacent |
| 59 | 33.197431 | 35.683367 | Grazing Pasture |  |
| 60 | 33.170555 | 35.6763 | Grazing Pasture |  |

**Supplementary Table 2**

Samples used in this research.

| # | Species/ Breed | Location of culling / Origin | Analysis | | | | |
| --- | --- | --- | --- | --- | --- | --- | --- |
|  |  |  | Gender | Mitochondrial sequence | Y sequence | STR analyses | Skull morphology |
| Jackie (17268) | *C. aureus* | Golan Heights | M | HCL1 | V | V | V |
| CA42 | *C. aureus* | Golan Heights | M | HCA1 | V | V |  |
| CA43 | *C. aureus* | Golan Heights | M | HCA1 | V | V |  |
| CA44 | *C. aureus* | Golan Heights | F | HCA1 |  |  |  |
| CA45 | *C. aureus* | Golan Heights | F | HCA1 |  |  |  |
| CA46 | *C. aureus* | Golan Heights | M | HCA4 | V | V |  |
| CA47 | *C. aureus* | Golan Heights | F |  |  |  |  |
| CA48 | *C. aureus* | Golan Heights | F |  |  |  |  |
| CA49 | *C. aureus* | Golan Heights | M |  | V | V |  |
| CA51 | *C. aureus* | Golan Heights | F | HCA4 |  |  |  |
| CA52 | *C. aureus* | Golan Heights | M |  | V |  |  |
| CA53 | *C. aureus* | Golan Heights | M | HCA3 | V | V |  |
| CA54 | *C. aureus* | Golan Heights | M | HCA4 | V | V |  |
| CA55 | *C. aureus* | Golan Heights | F | HCA4 |  | V |  |
| CA56 | *C. aureus* | Golan Heights | M |  | V |  |  |
| CA57 | *C. aureus* | Golan Heights | F | HCA1 |  | V |  |
| CA58 | *C. aureus* | Golan Heights | F | HCA1 |  | V |  |
| CA60 | *C. aureus* | Golan Heights | F |  |  |  |  |
| CA61 | *C. aureus* | Golan Heights | F |  |  |  |  |
| CA62 | *C. aureus* | Golan Heights | F |  |  |  |  |
| CA65 | *C. aureus* | Golan Heights | M | HCA1 | V | V |  |
| CA66 | *C. aureus* | Golan Heights | F | HCA4 |  | V |  |
| CA67 | *C. aureus* | Golan Heights | F | HCL1 |  | V |  |
| CA68 | *C. aureus* | Golan Heights | M | HCL1 |  |  |  |
| CA69 | *C. aureus* | Golan Heights | M | HCA4 |  |  |  |
| CA70 | *C. aureus* | Golan Heights | M | HCA4 |  |  |  |
| CA72 | *C. aureus* | Golan Heights | M | HCA4 |  | V |  |
| CA73 | *C. aureus* | Golan Heights | M | HCA4 |  |  |  |
| CA74 | *C. aureus* | Golan Heights | M | HCA4 |  |  |  |
| CA75 | *C. aureus* | Golan Heights | M | HCA4 |  |  |  |
| CA76 | *C. aureus* | Golan Heights | M | HCA5 |  |  |  |
| CA77 | *C. aureus* | Golan Heights | F | HCA4 |  |  |  |
| CA78 | *C. aureus* | Golan Heights | M | HCA4 |  |  |  |
| CA79 | *C. aureus* | Golan Heights | F | HCA4 |  |  |  |
| 16132 | *C. aureus* | Arava (south eastern desert) | M | HCA2 | V | V | V |
| 16142 | *C. aureus* | Dead Sea Area | M | HCA4 | V | V | V |
| 16172 | *C. aureus* | Negev | F | HCA1 |  | V | V |
| 15070 | *C. aureus* | Dead Sea Area | M | HCA1 | V | V |  |
| 15833 | *C. aureus* | Coastal plain | F |  |  |  | V |
| 15344 | *C. aureus* | Coastal plain | F | HCA1 |  |  |  |
| 15441 | *C. aureus* | Samaria (central israel) | M | HCA1 | V | V | V |
| 16190 | *C. aureus* | Coastal plain | F |  |  |  | V |
| 16180 | *C. aureus* | Negev | M | HCA1 | V | V | V |
| 16141 | *C. aureus* | Arava (south eastern desert) | M | HCA1 |  | V | V |
| 15561 | *C. aureus* | Coastal plain | M | HCL1 |  | V | V |
| 15440 | *C. aureus* | N/A | M | HCA1 |  | V | V |
| 16041 | *C. aureus* | Coastal plain | M | HCA1 |  | V |  |
| 16106 | *C. aureus* | Carmel Ridge | M | HCA1 |  | V | V |
| 16083 | *C. aureus* | Lower Galilee | M | HCA2 |  |  |  |
| 16213 | *C. aureus* | Negev | M | HCA1 |  |  |  |
| 15597 | *C. aureus* | Carmel Ridge | M | HCA1 |  |  |  |
| 15435 | *C. aureus* | Carmel Ridge | M | HCA1 |  |  |  |
| 15587 | *C. aureus* | Coastal plain | F |  |  |  | V |
| 17136 | *C. aureus* | Negev | M |  |  |  | V |
| 16118 | *C. aureus* | Lower Galilee | F |  |  |  | V |
| 115290 | *C. aureus* | Morphosource |  |  |  |  | V |
| 115292 | *C. aureus* | Morphosource |  |  |  |  | V |
| 114671 | *C. aureus* | Morphosource |  |  |  |  | V |
| 114633 | *C. aureus* | Morphosource |  |  |  |  | V |
| 16117 | *C.l. pallipes* | Judean Desert | F |  |  | V |  |
| 15800 | *C.l. pallipes* | Lower Galilee | F |  |  | V |  |
| 16234 | *C.l. pallipes* | Golan Heights | M |  |  | V | V |
| 13645 | *C.l. pallipes* | Mount Hermon | M |  |  | V | V |
| 16091 | *C.l. pallipes* | Carmel Ridge | F |  |  | V |  |
| 16115 | *C.l. pallipes* | Lower Galilee | F |  |  | V |  |
| 16238 | *C.l. pallipes* | Golan Heights | F |  |  | V | V |
| 15853 | *C.l. pallipes* | Arava (south eastern desert) | M | HCLP2 |  |  |  |
| 16093 | *C.l. pallipes* | Golan Heights | F | HCLP1 |  | V |  |
| 16105 | *C.l. pallipes* | Lower Galilee | F | HCLP1 |  |  |  |
| 16191 | *C.l. pallipes* | Golan Heights | F | HCLP1 |  | V |  |
| CL454 | *C.l. pallipes* | Golan Heights |  |  |  | V |  |
| 16115 | *C.l. pallipes* | Lower Galilee | F |  |  |  | V |
| 16853 | *C.l. pallipes* | Upper Galilee | M |  |  |  | V |
| 16845 | *C.l. pallipes* | Golan Heights | M |  |  |  | V |
| 117236 | *C. lupus* | Morphosource |  |  |  |  | V |
| 115894 | *C. lupus* | Morphosource |  |  |  |  | V |
| 23010 | *C. lupus* | Morphosource |  |  |  |  | V |
| 115946 | *C. lupus* | Morphosource |  |  |  |  | V |
| 115318 | *C. lupus* | Morphosource |  |  |  |  | V |
| 69 | Boxer | Private owner | F | HCLF1 |  |  |  |
| 202 | Cocker Spaniel | Breeder | F | HCLF2 |  | V |  |
| 17 | Labrador | Vet clinic |  |  |  | V |  |
| 20 | Border Collie | Vet clinic |  |  |  | V |  |
| 48 | Saint Bernard | Breeder |  |  |  | V |  |
| 55 | Canaan Dog | Private owner | F |  |  | V |  |
| 60 | Spanish Water Dog | Private owner | M |  |  | V |  |
| 61 | Spanish Water Dog | Private owner | F |  |  | V |  |
| 101 | Border Collie | Breeder | M |  |  | V |  |
| 102 | Sheltie | Breeder | F |  |  | V |  |
| 103 | Sheltie | Breeder | F |  |  | V |  |
| 104 | Border Collie | Breeder | F |  |  | V |  |
| 106 | Australian Sheperd | Breeder |  |  |  | V |  |
| 107 | Sarplaninac | Breeder | F |  |  | V |  |
| 108 | Sarplaninac | Breeder | M |  |  | V |  |
| 109 | Sarplaninac | Breeder | M |  |  | V |  |
| 57 | Australian cattle dog (blue heeler) | Private owner | F |  |  | V |  |
| 67 | Canaan Dog | Private owner |  |  |  | V |  |
| 68 | Czechoslovakian Wolfdog | Private owner | F |  |  | V |  |
| 72 | Huskey | Private owner | F |  |  | V |  |
| 201 | Saint Bernard | Breeder | F |  |  | V |  |
| 401 | Akbash | Golan Heights Cowboys | M |  |  | V |  |
| 402 | Akbash | Golan Heights Cowboys | M |  |  | V |  |
| 234 | *V. vulpes* | KUPRI |  |  |  |  | V |
| 235 | *V. vulpes* | KUPRI |  |  |  |  | V |
| 801 | *V. vulpes* | KUPRI |  |  |  |  | V |
| 16056 | *V. vulpes* | Galilee | F |  |  |  | V |
| 16067 | *V. vulpes* | Negev | M |  |  |  | V |
| 116030 | *V. vulpes* | Morphosource |  |  |  |  | V |
| 105334 | *V. vulpes* | Morphosource |  |  |  |  | V |
| 115816 | *V. vulpes* | Morphosource |  |  |  |  | V |
| 115162 | *V. vulpes* | Morphosource |  |  |  |  | V |

Remark: V symbol indicates the analysis type performed on each sample. F = female; M = male; N/A =Not Analyzed / Not available.

HCA1-5, HCL1, HCLP1-2 and HCLF1-2 identify mtDNA control region haplotypes found in this study and respectively corresponding to *C. aureus*, *C. lupaster*, *C. lupus pallipes* and *C. l. familiaris*.

**Supplementary Table 3**

Osteometric landmarks and their definitions.

| 1 | Anterior point of the incisive bone |
| --- | --- |
| 2 | Anterior end of the suture between nasal bones |
| 3 | Anterior point of the external sagittal crest |
| 4 | Posterior point of the external sagittal crest |
| 5 | Basion |
| 6 | Posterior nasal spine |
| 7 | Anterior palatine foramen |
| 8 | Anterior edge of canine alveolus, right side |
| 9 | Infraorbital foramen, right side |
| 10 | External point between P4 and M1, right side |
| 11 | The superior point suture between the zygomatic process of temporal bone and temporal process of zygomatic bone, right side |
| 12 | Most inferior point of the orbit, right side |
| 13 | Level of angulus oculi medialis, right side |
| 14 | Zygomatic process of frontal bone, right side |
| 15 | Anterior edge of canine alveolus, left side |
| 16 | Infraorbital foramen, left side |
| 17 | External point between P4 and M1, left side |
| 18 | The superior point suture between the zygomatic process of temporal bone and temporal process of zygomatic bone, left side |
| 19 | Most inferior point of the orbit, left side |
| 20 | Level of angulus oculi medialis, left side |
| 21 | Zygomatic process of frontal bone, left side |
| 22 | The posterior point suture between the zygomatic process of temporal bone and temporal process of zygomatic bone (proc. temporalis ossis zygomaticus), right side |
| 23 | Asterion, posterior at occipital-parietal-temporal suture, right side |
| 24 | The posterior point suture between the zygomatic process of temporal bone and temporal process of zygomatic bone (proc. temporalis ossis zygomaticus), left side |
| 25 | Asterion, posterior at occipital-parietal-temporal suture, left side |
| 26 | Maxillary palatine suture at midline |
| 27 | Internal point between P4 and M1, right side |
| 28 | Internal point between P2 and P3, right side |
| 29 | Internal point between P4 and M1, left side |
| 30 | Internal point between P2 and P3, left side |
